# Novel intragenic deletion within the *FXN* gene in a patient with typical phenotype of Friedreich ataxia: may be more prevalent than we think?

**DOI:** 10.1186/s12920-023-01743-0

**Published:** 2023-12-01

**Authors:** Cinthia Aguilera, Anna Esteve-Garcia, Carlos Casasnovas, Valentina Vélez-Santamaria, Laura Rausell, Pablo Gargallo, Javier Garcia-Planells, Pedro Alía, Núria Llecha, Ariadna Padró-Miquel

**Affiliations:** 1 Genetics Laboratory, Laboratori Clínic Territorial Metropolitana Sud. Hospital Universitari de Bellvitge, Institut d’Investigació Biomèdica de Bellvitge (IDIBELL), L’Hospitalet de Llobregat, Spain; 2 Clinical Genetics Unit, Laboratori Clínic Territorial Metropolitana Sud. Hospital Universitari de Bellvitge, Institut d’Investigació Biomèdica de Bellvitge (IDIBELL), L’Hospitalet de Llobregat, Spain; 3 Neuromuscular Unit, Neurology Department, Hospital Universitari de Bellvitge, Institut d’Investigació Biomèdica de Bellvitge (IDIBELL), L’Hospitalet de Llobregat, Spain; 4 Neurometabolic Diseases Group, Institut d’Investigació Biomèdica de Bellvitge (IDIBELL), L’Hospitalet de Llobregat, Spain; 5 Biomedical Research Network Centre in Rare Diseases (CIBERER), Madrid, Spain; 6 Health in Code S.L, Valencia, Spain

**Keywords:** Friedreich ataxia (FRDA), *FXN*, Deletion, Biallelic expansion, Parental testing

## Abstract

**Background:**

Friedreich ataxia is the most common inherited ataxia in Europe and is mainly caused by biallelic pathogenic expansions of the GAA trinucleotide repeat in intron 1 of the *FXN* gene that lead to a decrease in frataxin protein levels. Rarely, affected individuals carry either a large intragenic deletion or whole-gene deletion of *FXN* on one allele and a full-penetrance expanded GAA repeat on the other allele.

**Case presentation:**

We report here a patient that presented the typical clinical features of FRDA and genetic analysis of *FXN* intron 1 led to the assumption that the patient carried the common biallelic expansion. Subsequently, parental sample testing led to the identification of a novel intragenic deletion involving the 5’UTR upstream region and exons 1 and 2 of the * FXN* gene by MLPA.

**Conclusions:**

With this case, we want to raise awareness about the potentially higher prevalence of intragenic deletions and underline the essential role of parental sample testing in providing accurate genetic counselling.

**Supplementary Information:**

The online version contains supplementary material available at 10.1186/s12920-023-01743-0.

## Background

Friedreich ataxia (FRDA; MIM# 229300) is the most common hereditary ataxia with an estimated prevalence of 1 in 21000 Europeans and a carrier frequency of approximately 1 in 70 [1]. The main clinical characteristic of the condition is the gradual loss of limb and gait coordination with onset usually before the age of 25 years [2]. However, 1 in 4 individuals with FRDA will display symptoms later in life, after age 50 [3]. Clinical manifestations of FRDA also include progressive muscle weakness, spasticity, dysarthria, and loss of joint position or vibration sense [4]. Less frequently, patients with FRDA can experience hypertrophic cardiomyopathy, diabetes mellitus, scoliosis, *pes cavus* and vision loss [3].

FRDA is caused by a deficiency of a mitochondrial protein called frataxin, encoded by the *FXN* gene located on chromosome 9. Frataxin is thought to be involved in iron metabolism participating in processes such as iron storage and assembling clusters of iron and sulfur molecules, among others [5, 6]. Its deficiency leads to mitochondrial dysfunction as a result of iron accumulation, reduced ATP production and oxidative stress due to an increase in the production of reactive oxygen species (ROS) [7].

The main molecular mechanism underlying the decrease in frataxin levels is biallelic pathogenic expansions of the GAA trinucleotide repeat in intron 1 of the *FXN* gene, accounting for 96% of affected FRDA individuals [8]. However, to a lesser extent, affected individuals are compound heterozygous for a full penetrance expanded GAA repeat and a single pathogenic nucleotide variant. More rarely, affected individuals carry either a large intragenic deletion or whole-gene deletion of *FXN* on one allele and a full-penetrance expanded GAA repeat on the other allele [9, 10].

All patients with a clinical diagnosis of FRDA undergo fragment analysis of the polymorphic GAA region of the first intron of the *FXN* gene, which allows the measurement of the number of GAA repeats [4]. Based on allele size, three categories can be distinguished: (i) normal alleles, those ranging from 5 to 33 GAA repeats, (ii) intermediate alleles consisting of those ranging from 34 to 65 repeats and (iii) pathogenic alleles, those higher than 66 repeats. However, this method would not detect deletions/duplications, nor single nucleotide variants (SNVs) [9].

FRDA is an autosomal recessive condition. The parents of an affected individual are obligated heterozygous carriers of a pathogenic *FXN* variant (a pathogenic expanded allele, a pathogenic SNV or a deletion/duplication). *De novo* expansion prevalence is unknown although it is thought to be very rare. Parental sample testing is essential to provide an accurate risk assessment and offer carrier testing to other at-risk family members.

Here, we describe a novel intragenic deletion identified in a 32-year-old patient with classical clinical features of FRDA. The patient was initially assumed to carry the common biallelic pathogenic expanded GAA repeat in intron 1 of the *FXN* gene. With this case, we want to highlight that it is possible that intragenic deletions may be more prevalent than previously thought and the importance of parental testing in order to provide appropriate genetic counselling.

## Case presentation

### Clinical course

The proband is a 32-year-old male who was diagnosed with FRDA at the age of 23, after 5 years of progressive evolution of gait and limb ataxia and mild dysarthria. Neurological examination showed hypotonia, dysmetria, dyschronometry, dysdiadochokinesia, Stewart Holmes sign in all four extremities, hypopalesthesia and arthro-kinetic hypoesthesia, positive Romberg sign, flexor plantar reflexes and universal areflexia. He also presented scoliosis, *pes cavus* and muscle weakness. Cranial and spinal Magnetic Resonance Imaging (MRI) were normal. He did not have hearing, vision nor endocrine manifestations and cardiovascular examination was unremarkable. As FRDA was suspected, genetic analysis of *FXN* gene intron 1 was performed in another laboratory showing a biallelic GAA repeat expansion and confirming the clinical diagnosis.

Currently, the patient has not developed any cardiac, endocrine nor optic symptoms. He was referred to Clinical Genetics Unit for at-risk carrier testing and parental testing showed some inconsistencies.

### Analysis of ***FXN*** gene

Parental and sibling’s DNA samples were extracted from peripheral blood using an automatable extraction method (Maxwell RSC Whole Blood DNA kit, Promega, Wisconsin, USA) following manufacturer’s instructions. Polymorphic region in intron 1 of the *FXN* gene containing the GAA triplet expansion was amplified by PCR according to the procedure described by Campuzano et al., 1996 [8]. Coriell sample NA16216 (biallelic GAA expansion of 200 and 500 repeats) was used as a positive control (Supplementary Fig. S1). To detect expanded alleles Triplet Repeat Primed PCR (TP-PCR) was carried out using the primers and the conditions described by Ciotti et al., 2004 [11]. Amplification products were run on a 3500 Genetic Analyzer (Applied Biosystems, California, USA) using as a size standard GeneScan™ 600 LIZ™ Dye. Peak sizes were determined with Gene Marker v1.90 software (Softgenetics, Pennsylvania, USA).

**Fig. 1:**
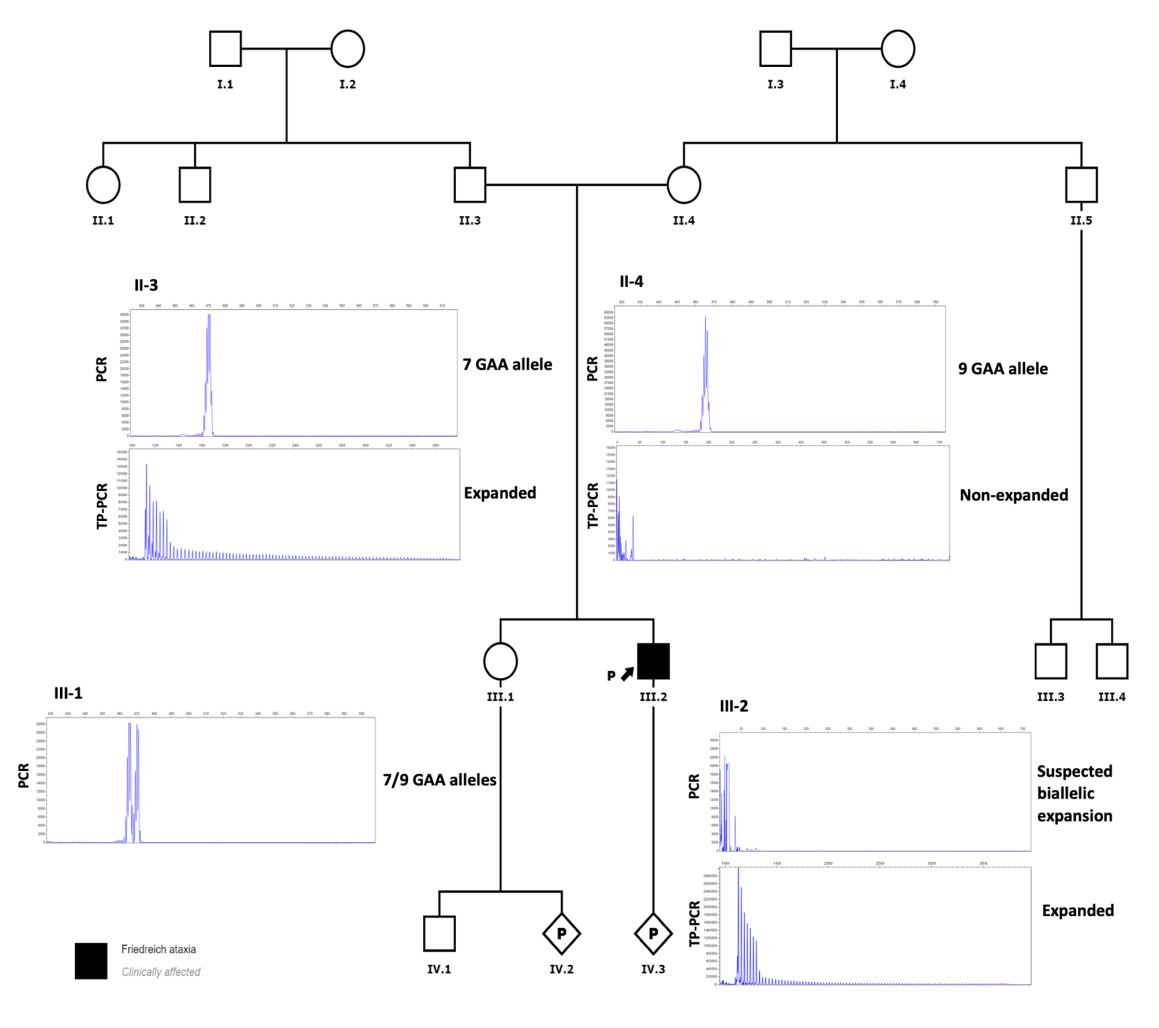
Pedigree and genetic analysis of the *FXN* polymorphic region performed on the proband and his family. The results of PCR fragment analysis show an allele of 7 GAA repeats in the father’s sample (II-3) and an allele of 9 GAA repeats in the mother’s sample (II-4). The sibling (III-1) presents two normal alleles of 7 and 9 GAA repeats, respectively. No peaks were observed in the proband’s sample (III-2). To detect expanded alleles, TP-PCR was performed and, as a result, the father (II-3) and the proband’s (III-2) samples showed an expansion while non-expanded alleles were observed in the mother’s sample (II-4). The affected individuals are marked in black

PCR fragment analysis showed that the paternal sample (II-3) was carrying an allele of 7 GAA repeats and an expansion was observed by TP-PCR, corresponding to a healthy carrier of the disease. On the other hand, the maternal sample (II-4) only showed a peak corresponding to 9 GAA repeats but no expansion was observed. The proband’s sibling (III-1) had two normal alleles of 7 and 9 GAA repeats (Fig. 1). The mother’s result did not correlate with a biallelic expansion in the proband. Therefore, a new DNA sample was obtained from the proband in order to repeat the genetic analysis of FRDA, which showed no peaks in the PCR fragment analysis although a band at the same level as the positive control (NA16216) was observed in the agarose gel (Supplementary Fig. S1). Proband’s TP-PCR showed an expansion, suggesting that the patient may carry a biallelic expansion, which was in agreement with the first genetic test performed in the patient (Fig. 1).

The lack of an expansion in the mother and the fact that no normal allele was observed in the proband’s PCR fragment analysis led us to hypothesize the presence of a deletion. Subsequently, duplication/deletion analysis of *FXN* gene was performed using the Multiplex Ligation-dependent Probe Amplification (MLPA) Probemix P316-B4 Recessive Ataxias kit (MRC Holland, Amsterdam, The Netherlands). The kit contains five probes, one for each exon of the *FXN* gene (NM_000144.5, 9q21.11). The ligation site of the first *FXN* probe is located 232 nucleotides before exon 1. MLPA was carried out according to manufacturer’s instructions (MRC Holland) and amplification products were run on 3500 Genetic Analyzer (Applied Biosystems). Coffalyser.Net v.220513.1739 (MRC Holland) software was used to analyze MLPA results.

**Fig. 2:**
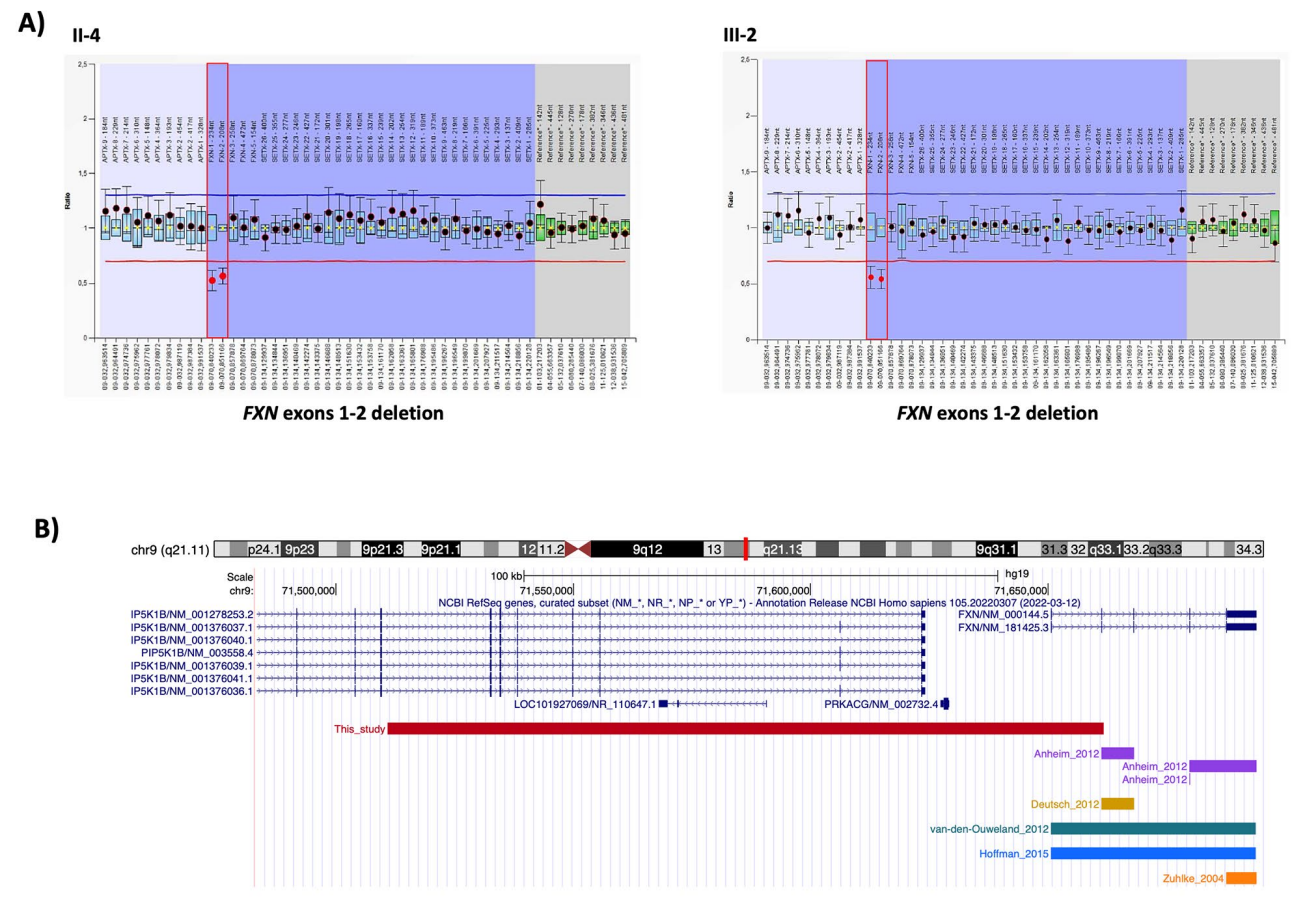
Deletion/duplication analysis of *FXN* gene and summary of intragenic deletions reported to date. **(A)** MLPA analysis with the kit P316-B4 showed a deletion of *FXN* exons 1 and 2 in the mother (II-4) and the proband’s (III-2) samples. The deleted probes are highlighted in red. **(B)** Summary of *FXN* gene deletions in the literature and this report

A reduction of 50% in the relative peak height of the probes corresponding to *FXN* exons 1 and 2 (NC_000009.11:g.(?_71650436)_(71661379_71668090)del) was observed, while the probes corresponding to *FXN* exons 3, 4 and 5 showed a normal diploid dosage (Fig. 2A). So, it was concluded that the mother carried an allele of 9 GAA repeats and a deletion spanning the 5’UTR (the probe that corresponds to exon 1 is located 232 nucleotides upstream of exon 1) as well as exons 1–2 of *FXN* gene. On the other hand, the proband had an expansion inherited from the father and a deletion encompassing 5’UTR and exons 1–2 of *FXN* gene of maternal origin. It is worth noting that the size of the expanded allele could not be determined with the methods used in this study. An option for confirming the shared allele between the father and the son would be to either conduct a paternity test or verify the size of the shared allele through alternative means.

In order to determine the breakpoints of the deletion whole genome sequencing (WGS) was performed using xGen DNA Library Prep Kit EZ (Integrated DNA Technologies, Iowa, USA). The subsequent library was sequenced on a NovaSeq 6000 S4 instrument (Illumina, San Diego, USA). Reads were aligned to GRCh37 build and variant calling was performed in agreement with GATK guidelines [12]. Copy number variants were called using DRGN-MANTA v3.9 (Illumina, San Diego, USA). The results showed that the deletion spans 151,09 kb (chr9:71,510,734–71,661,828) and includes exons 9–16 of the *PIP5K1B* gene (NM_003558.4), the entire *PRKACG* gene (NM_002732.4) and exons 1–2 of the *FXN* gene (NM_000144.5) (Fig. 2B).

## Discussion and conclusions

Friedreich ataxia is the most common inherited ataxia in Europe [13]. The main mechanism of the disease, in 96% of the cases, is biallelic GAA repeat expansion in the *FXN* gene. However, there are other mechanisms that are less frequent which include intragenic pathogenic variants or deletion/duplications in combination with a GAA expanded allele in the *FXN* gene [2]. When there is a clinical suspicion of FRDA, the recommended molecular test includes the analysis of the polymorphic region of intron 1 of *FXN* gene by PCR and TP-PCR. These techniques lack the detection of pathogenic SNVs and deletions/duplications in the *FXN* gene. Individuals that are heterozygous for a GAA expansion and present a consistent FRDA phenotype undergo whole *FXN* gene sequencing. Additionally, when no pathogenic/likely pathogenic variants are identified other methods are applied such as quantitative analysis on frataxin copy number by MLPA [14].

The absence of normal alleles in the PCR and the detection of an expansion by TP-PCR should not lead to the assumption that the individual carries a biallelic expansion, as intragenic deletions may be more common than expected. To date, 10 patients carrying intragenic deletions in the *FXN* gene have been reported in the literature [9, 15–18] (Fig. 2B).

Genotype-phenotype correlation of individuals carrying intragenic deletions in combination with a GAA expansion on the other allele showed an earlier onset of the disease, rapid progression of cardiological and neurological manifestations, and severe hypertrophic cardiomyopathy compared with patients presenting a biallelic expansion [9].

Here, we report a novel intragenic deletion that includes the 5’UTR upstream region and exons 1 and 2 of the* FXN* gene. This unreported deletion is predicted to eliminate the start codon, and consequently no protein will be produced (NP_000135.2:p.0?). Loss of function is a known disease-causing mechanism for this condition, so the deletion was classified as pathogenic. Conversely, the expanded allele will lead to a reduction in *FXN* transcript levels due to transcriptional silencing, ultimately resulting in a deficiency of frataxin levels [2]. In contrast to other patients heterozygous for an expansion and an exonic deletion, our patient presents the typical features of FRDA, the onset of the disease was at the age of 23, the progression is as expected for the disease and he does not have cardiomyopathy (Table 1). The delay in age of onset could potentially be attributed to interruptions in the 3’ region of the GAA repeat tract, as evidenced by a signal drop in the TP-PCR assay and according to the study conducted by Nethisinghe et al., 2021 [19]. Other factors such as modifying genes, environmental factors, or somatic mosaicism may also contribute to the differences in disease severity.

Parental sample testing was essential in the case presented, as we were able to detect that the mother and the proband were carriers of an intragenic deletion not reported before. This finding also has implications for other family members who could be possible carriers of the deletion and taking into account the elevated carrier frequency of FRDA (1/70), they could be at high risk of having children with FRDA. This is the reason why we suggest that, if available, parental sample testing should be performed and not assume that parents are obligate carriers of an expansion since it is possible that deletions may be more prevalent than it was initially thought.

In conclusion, we describe a patient presenting with novel intragenic deletion and an expansion on the *FXN* gene who shows the typical progression and clinical features of FRDA. We believe that parental sample testing should be performed in all FRDA patients that present an apparent biallelic expansion in order to offer proper genetic counselling.

**Table 1:** Clinical features of the patient according to what is described for typical FRDA manifestations

Clinical features associated with Friedreich Ataxia†	Patient with expansion / deletion of 5’UTR and exons 1–2 in the *FXN* gene
Onset before age 25 years	+
Dysarthria	+
Decrease in/loss of position sense and/or vibration sense in lower limbs	+
Pyramidal weakness of the legs	+
Extensor plantar response	Indifferent
Muscle weakness	+
Progressive afferent and cerebellar ataxia of gait and all four limbs	+
Scoliosis	+
Pes cavus	+
Nystagmus	-
Cardiomyopathy	-
Glucose intolerance	-
Diabetes mellitus	-
Optic atrophy and/or deafness	-

†Clinical features of Friedreich ataxia described by Bidichandani et al., 1993 and Corben et al., 2014. +, present; -, not present

### Electronic supplementary material

Below is the link to the electronic supplementary material.

Supplementary Material 1

Supplementary Material 2

## Data Availability

The data generated as part of this study is available on request to the corresponding author. The dataset generated and/or analysed during the current study are available in the European Variation Archive (EVA) repository [accession number PRJEB65722, https://www.ebi.ac.uk/eva/?eva-study=PRJEB65722].

## List of abbreviations

**FRDA**: Friedreich ataxia
***FXN***: frataxin
**PCR**: polymerase chain reaction
**TP-PCR**: Triplet Repeat Primed PCR
**MLPA**: Multiplex Ligation-dependent Probe Amplification
