# Supplementary material for: Novel intragenic deletion within the FXN gene in a patient with typical phenotype of Friedreich ataxia: may be more prevalent than we think?
Source: BMC Med Genomics. 2023 Dec 1;16:312. doi: 10.1186/s12920-023-01743-0 (PMC10693098; doi:10.1186/s12920-023-01743-0)
Supplement: Supplementary file 1 — Supplementary Material 1 [file 12920_2023_1743_MOESM1_ESM.pdf]

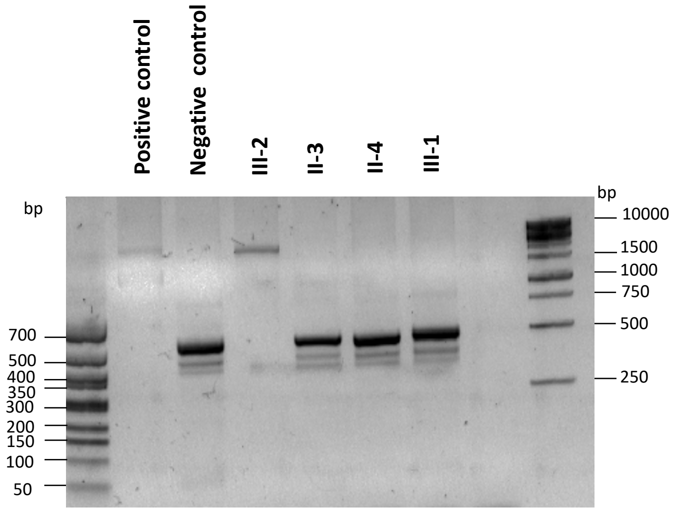

Supplementary Fig. S1. Agarose gel showing the results of the PCR amplifying the polymorphic region of *FXN* intron 1. A band could be observed in the proband's sample (III-2) at the same level as the positive control corresponding to an expansion. The father (II-3), mother (II-4) and sibling's (III-1) samples showed a band at the same level as the negative control. The non-expanded allele was preferentially amplified in the father's sample (II-3), resulting in the inability to observe the expanded allele. The agarose gel was cropped to remove the empty lanes and the original image could be found as supplementary material S2.
