# Supplementary figures and images for: Novel intragenic deletion within the FXN gene in a patient with typical phenotype of Friedreich ataxia: may be more prevalent than we think?
Source: BMC Med Genomics. 2023 Dec 1;16:312. doi: 10.1186/s12920-023-01743-0 (PMC10693098; doi:10.1186/s12920-023-01743-0)

**Supplementary Fig. S2.** The original images of the agarose gel.

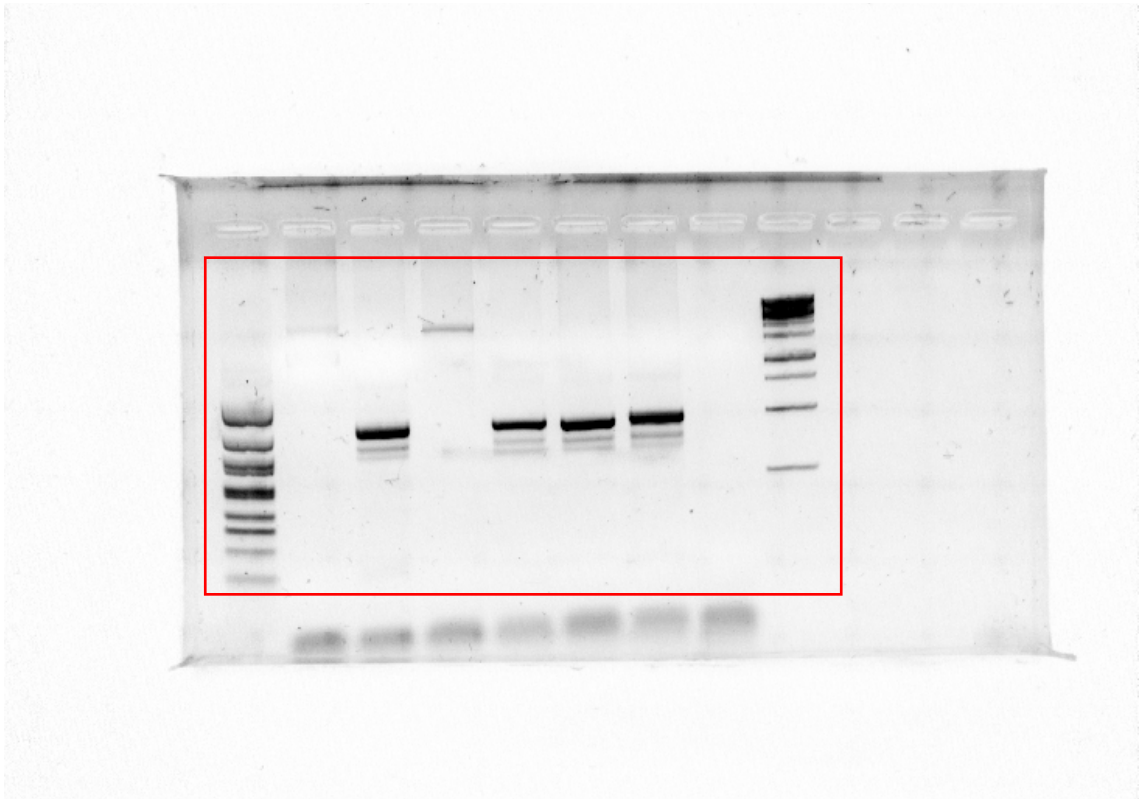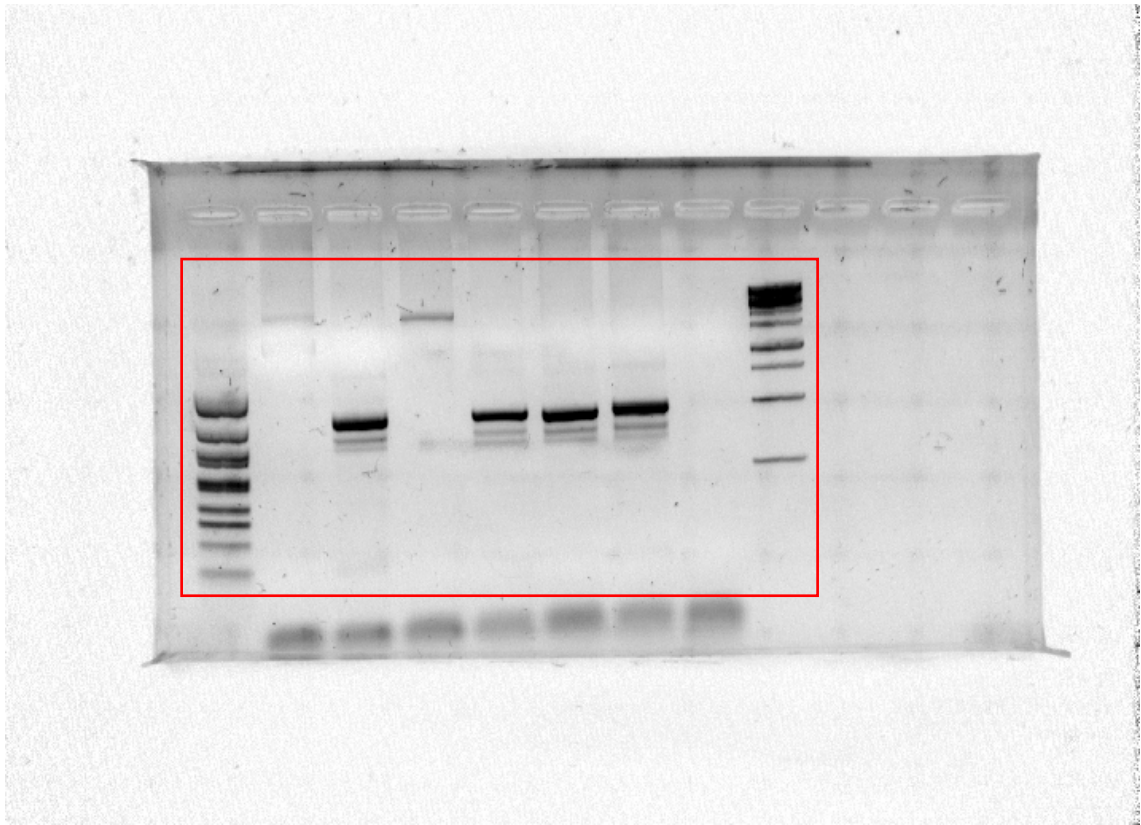

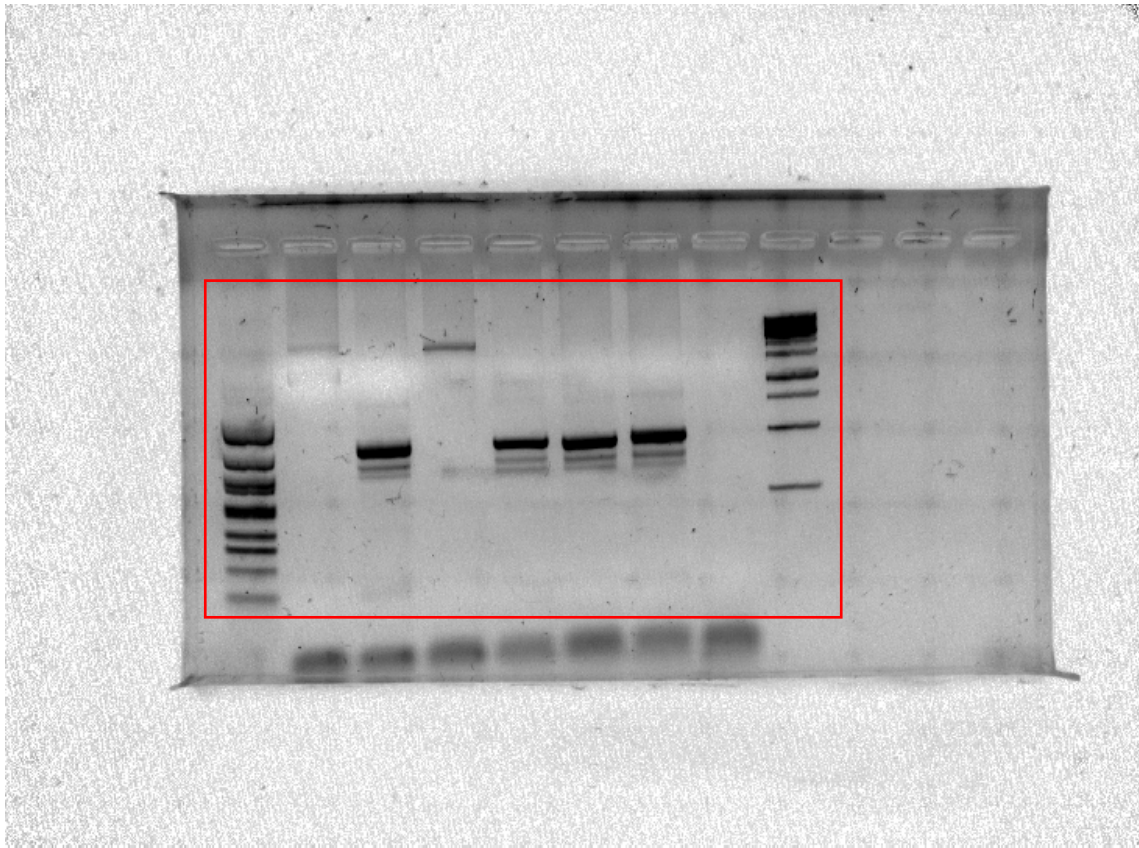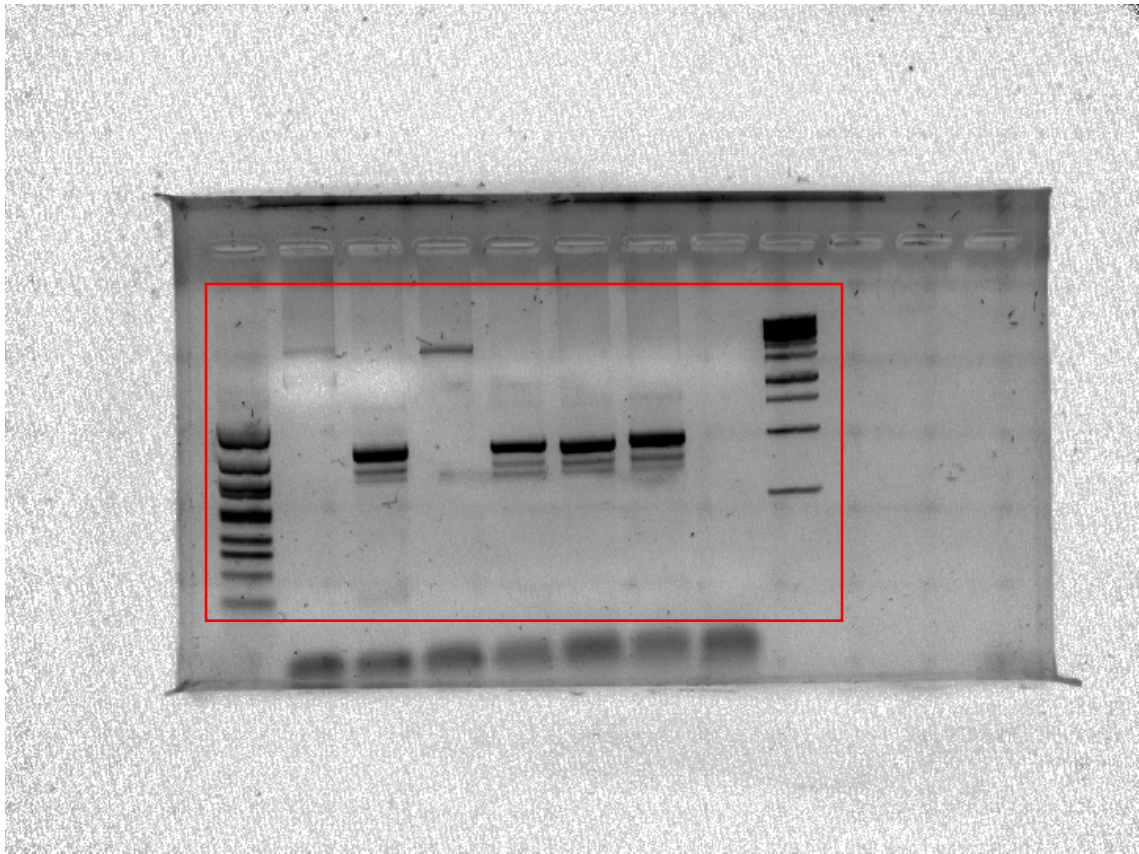

Supplement: Supplementary file 2 — Supplementary Material 2 [file 12920_2023_1743_MOESM2_ESM.pdf]
